# Circadian Rhythm Sleep Disorders: Genetics, Mechanisms, and Adverse Effects on Health

**DOI:** 10.3389/fgene.2022.875342

**Published:** 2022-04-29

**Authors:** Chaoran Liu, Xiangrong Tang, Zishan Gong, Wang Zeng, Qiao Hou, Renbin Lu

**Affiliations:** ^1^ Xinjiang Medical University, Urumqi, China; ^2^ Center for Medical Genetics, School of Life Sciences, Central South University, Changsha, China; ^3^ Department of Rehabilitation Medicine, Xiangya Third Hospital, Central South University, Changsha, China; ^4^ Hunan Key Laboratory of Molecular Precision Medicine, Xiangya Hospital, Central South University, Changsha, China; ^5^ National Clinical Research Center for Geratric Disorder, Xiangya Hospital, Central South University, Changsha, China

**Keywords:** circadian clock, genetics, mutations, sleep, circadian rhythm sleep disorders

## Abstract

Nearly all living organisms, from cyanobacteria to humans, have an internal circadian oscillation with a periodicity of approximately 24 h. In mammals, circadian rhythms regulate diverse physiological processes including the body temperature, energy metabolism, immunity, hormone secretion, and daily sleep-wake cycle. Sleep is tightly regulated by circadian rhythms, whereas a misalignment between the circadian rhythms and external environment may lead to circadian rhythm sleep disorders (CRSD). CRSD includes four main kinds of disorders: the advanced sleep-wake phase disorder (ASPD), the delayed sleep-wake phase disorder (DSPD), the irregular sleep-wake rhythm disorder and the non-24-h sleep-wake rhythm disorder. Recent studies have begun to shed light on the genetic basis of CRSD. Deciphering the genetic codes for ASPD and DSPD has so far been more successful than the other CRSDs, which allow for the development of animal models and understanding of the pathological mechanisms for these disorders. And studies from humans or animal models implicate CRSDs are associated with adverse health consequences, such as cancer and mental disorders. In this review, we will summarize the recent advances in the genetics, underlying mechanisms and the adverse effects on health of ASPD and DSPD.

## Introduction

Sleep is fundamental to the health of human, and remains one of the greatest mysteries in science. The timing, depth, and duration of sleep is regulated by the circadian system (termed process C) and the sleep homeostat (termed process S), which is known as the two-process model (Borbely, 1982). Process S reflects how sleep pressure accumulates during wakefulness and is discharged during sleep. It operates like an internal timer that measures the tendency to fall asleep when the subject is awake and the tendency to wake up when the subject is asleep. Process C (i.e. circadian clock) functions to restrict sleep within a time of day that is ecologically appropriate (Ashbrook, Krystal et al., 2020). In mammals, the suprachiasmatic nucleus (SCN) at the hypothalamus is the pacemaker of circadian clocks. After SCN lesion, the circadian rhythm in the sleep-wake cycle is completely eliminated, although ultradian rhythms of 2–4 h periodicity persist (Eastman, Mistlberger et al., 1984). The SCN also receives direct retinal input via the retinohypothalamic tract (RHT), which enable the central clock to entrain to external light/dark cues (Hastings, Maywood et al., 2018). According to the two-process model, it is the interaction of process C and process S that determines when we wake and sleep (Franken & Dijk, 2009).

Sleep is tightly regulated by the circadian rhythms. In optimal conditions, circadian rhythm is aligned with light/dark cycle, work, family, and social obligations (Mahowald & Schenck, 2005). However, environmental light/dark cycle changes (such as shift work, jet lag, nighttime light exposure etc.) and/or genetic abnormalities impair proper entrainment of the circadian system, resulting in chronic circadian rhythm sleep disorder (CRSD) (Sehgal & Mignot, 2011; Haus & Smolensky, 2013; Khosravipour, Khanlari et al., 2021). Accumulated evidences suggest that CRSD may be detrimental to physical health and mental function, with increase in the incidence of obesity, cancer, metabolic syndrome, cardiovascular diseases and metal disorders (Kettner et al., 2016; Musiek & Holtzman, 2016; Papantoniou et al., 2018; Rijo-Ferreira & Takahashi, 2019; Sancar and Van Gelder, 2021).

In recent years, great progress has been made in deciphering the genetic basis for CRSD, such as ASPD and DSPD. Based on these discoveries, reliable animal models have been established to understand the pathological mechanisms as well as the potential adverse influences for these disorders. Intriguingly, studies on the CRSD-related human genetics also improve our understanding in the operating mechanisms underlying circadian clock.

### The Molecular Mechanism of Circadian Rhythms

Circadian rhythms are endogenous biological processes, through which, all organisms can predict and adapt to the environmental changes corresponding with the day-night cycle and adjust their physiological functions and behaviors accordingly (Song & Rogulja, 2017). Light is the main cue for the entrainment of circadian rhythms to the external environment, and the SCN functions as a pacemaker responsible for this coordination process. Moreover, circadian clock can be also entrained by hormones, body temperature or feeding/fasting (Claustrat, Brun et al., 2005; Asher & Sassone-Corsi, 2015). Actually, it turns out that circadian clocks exist in almost all cells and tissues in our body (Dibner, Schibler et al., 2010; Koronowski & Sassone-Corsi, 2021).

**FIGURE 1:**
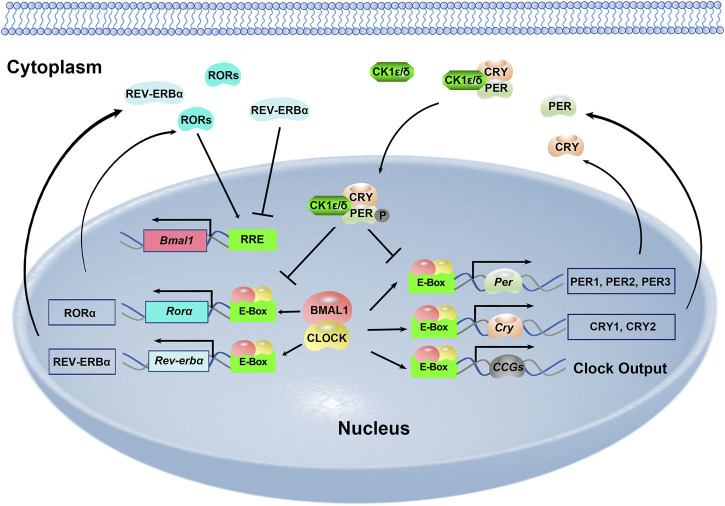
Molecular framework of the mammalian circadian clock.

The mammalian circadian clock is fundamentally based on the transcriptional-translational feedback loops (Figure 1). At the core of this molecular network are two transcription factors: circadian locomotor output cycles kaput (CLOCK) and brain and muscle aryl hydrocarbon receptor nuclear translocator-like 1 (BMAL1). They heterodimerize and bind to E-box elements (CACGTG) located at the promoters of clock genes as well as a large number of clock-controlled genes (CCGs). This mechanism drives the expression of Period genes (Per1–3) and Cryptochrome genes (Cry1/2). PER and CRY proteins gradually accumulate in the cytoplasm and PER proteins are phosphorylated by casein kinase Iδ (CKIδ) and CKIε. PER, CRY and CKI proteins form a complex and translocate to the nucleus to inhibit the transcriptional activity of the CLOCK-BMAL1. This negative-feedback loop takes approximately 24 h to complete. Meanwhile, there are additional feedback loops driven by CLOCK: BMAL1. RORα promotes while REV-ERBα inhibits Bmal1 transcription via binding to the ROR element (RRE) motif on the Bmal1 promoter (Figure 1). And DEC1 and DEC2 are two suppressors for CLOCK-BMAL1 heterodimer. The transcription of Rorα, Rev-erbα, Dec1 and Dec2 is positively regulated by CLOCK-BMAL1, and negatively regulated by PER1, PER2, CRY1 and CRY2 (Honma, Kawamoto et al., 2002; Patke, Young et al., 2020; Takahashi, 2017; Ueda, Hayashi et al., 2005).

### Circadian Rhythm Sleep Disorders

Circadian rhythm sleep disorders (CRSDs) are conditions that the internal circadian rhythms are not properly aligned with the external environment. CRSDs are divided into four main types, including advanced sleep phase disorder (ASPD), delayed sleep phase disorder (DSPD), irregular sleep-wake rhythm and non-24-h sleep-wake disorder. Although the environmental, social, and/or occupational schedules may lead to sleep disturbance, some individuals may also be genetical predisposed to the development of CRSD (Chong, Ptacek et al., 2012). It is estimated that ∼33% of sleep quality variance and ∼40% of sleep pattern variance are contributable to genetic differences (Heath, Kendler et al., 1990).

### Advanced Sleep Phase Disorder

Individuals with advanced sleep phase disorder usually feel very sleepy and have to go to bed early in the evening (generally between 6–9 pm) and wake up very early in the morning (generally between 2–5 am). The sleep, temperature, and melatonin rhythms shift forward 3–4 h as compared to the normal persons (Jones, Campbell et al., 1999). However, their sleep quality and duration are normal as average peoples when they are allowed to sleep at their desired times (Ito & Inoue, 2015).

ASPD is a rare disorder with a strong genetic trait. The research team led by Louis J Ptáček and Ying-Hui Fu at the University of California at San Franscisco (UCSF) have made great contributions to decipher the underlying genetic mechanisms of ASPD. In 1999, Jones et al. reported three families with ASPD, which show autosomal dominant inheritance (Jones et al., 1999). In 2001, Toh et al. identified an S662G mutation in hPer2, located near the telomere of chromosome 2q, as the causative mutation for one of these ASPD pedigrees (Table 1). Ser662 is located within the CKIε binding region in PER2, and S662G mutation leads to decreased phosphorylation of PER2 *in vitro* (Toh, Jones et al., 2001). It should be noted that the tau mutant hamsters with short-period behavioral rhythms (∼20 h in homozygotes, and ∼22 h in heterozygotes) have a missense mutation in the CKIε gene.

**TABLE 1:** Genes implicated in human circadian rhythm sleep disorders.

Sleep Disorder	Gene	Mutation	Phenotypes in transgenic mice	References
Advanced Sleep Phase Disorder (ASPD)	Per2	S662G	Advanced Phase of circadian activity; ∼2 h shorter Period (τ)	Toh et al. (2001)
				Xu et al. (2007)
	CK1δ	T44A	A shorter circadian Period (τ)	Xu et al. (2005); Brennan et al. (2013)
		H46R	\	Brennan et al. (2013)
	Cry2	A260T	Advanced Phase of circadian activity; A shorter circadian Period (τ)	Hirano et al. (2016)
	Per3	P415A/H417R	Mild depression-like phenotype; Longer circadian behavioral period; ∼4-h phase delay in light-dark cycles (LD 4:20)	Zhang et al. (2016)
Delayed sleep phase disorder (DSPD)	Per3	Polymorphisms	\	Pereira et al. (2005); Archer et al. (2010)
	Cry1	c.1657+3A > C	\	Patke et al. (2017); Onat et al. (2020)

Although phosphorylation of PER2 by CKIε retards the nuclear entry of PER2(Vielhaber, Eide et al., 2000) and decreases the stability of PER2 protein (Keesler, Camacho et al., 2000), S662G mutation does not affect PER2 degradation or nuclear localization (Xu, 2007). Interestingly, studies from Xu et al. suggest that Ser662 of PER2 is not phosphorylated by CKIε; however, a phosphate at S662 is required for CKIε to phosphorylate other residues in PER2. They further generated transgenic mice carrying S662G hPER2 gene, which faithfully recapitulate the ASPD phenotype in human (Xu, Toh et al., 2007).

In 2005, Xu et al. found a T44A mutation in human CKIδ gene co-segregates with the ASPD phenotypes in another pedigree (Table 1) (Xu, Padiath et al., 2005). The T44A mutant kinase has significantly lower enzymatic activity than wild-type kinase. Both drosophila and mice carrying the T44A hCKIδ exhibit abnormal circadian rhythms. Transgenic mice carrying T44A hCKIδ show a shorter circadian period, recapitulating the ASPD phenotype in human; however, the T44A hCKIδ transgenic flies show a longer circadian period, suggesting divergent regulatory mechanisms in mammalian and fly clocks. Although transgenic mice carrying wild-type (WT) CKIδ have abnormal circadian period, WT CKIδ transgene further shortens the circadian period in the S662G hPER2 transgenic mice, indicating that CKIδ may regulate circadian period through PER2 *in vivo*.

In 2016, Hirano et al. identified a missense mutation (A260T) in hCry2 gene that is associated with ASPD (Table 1). The Ala260 is located in the flavin adenine dinucleotide (FAD) binding domain of CRY2 protein, and the A260T mutation alters the conformation of CRY2 protein and increases its affinity to the E3 ubiquitin ligase FBXL3, thus promoting the degradation of CRY2. The transgenic mice carrying hCRY2-A260T have advanced phase of sleep-wake behavior in a light-dark cycle and a shortened circadian period in constant darkness, mimicking the ASPD phenotype in human (Hirano, Shi et al., 2016).

Zhang et al. identified two rare variants in PER3 (P415A and H417R on the same allele) in ASPD patients accompanied with increased depressive mood and global seasonality scores (Table 1). P415A/H417R-PER3 is less stable and has reduced repressive activity than WT PER3. In addition, this mutation fails to stabilize PER1/2 proteins as WT PER3. The circadian period of hPER3-P415A/H417R transgenic mice under constant light (LL) is significantly longer than the controls, and hPER3-P415A/H417R transgenic mice show a ∼4-h phase delay in activity onset and offset time under 4-h light/20-h dark cycles versus controls, which seems contradict to the APSD in human. Yet, flies expressing hPER3-P415A/H417R show significantly earlier activity offset time under light-dark cycle (LD) and shorter circadian period under constant darkness (DD) as compared with hPER3-WT flies, which recapitulate the APSD in human (Zhang et al., 2016).

Mammalian Timeless (mTim) is as a homolog of Drosophila Timeless (dTim) (Koike, Hida et al., 1998), which is a core component of the Drosophila clock and functions as negative regulator necessary for generating rhythmicity and photoentrainment in flies (Engelen, Janssens et al., 2013). Conditional knockdown of mTim protein expression in the rat SCN disrupted SCN neuronal activity rhythms and altered levels of known core clock elements (Barnes, Tischkau et al., 2003). Recently, the Kurien et al. identified an ASPD-associated TIM-R1081X mutation by using unbiased whole-exome sequencing. The TIM-R1078X knock-in mice exhibit FASP phenotype with altered photic entrainment but normal circadian period. Furthermore, the TIM-R1078X variant lead to a decrease of TIM accumulation in the nucleus and affinity for CRY2, resulting in destabilization of PER/CRY complex and a shortened period in mouse embryonic fibroblasts (Kurien, Hsu et al., 2019).

### Delayed Sleep Phase Disorder

Delayed sleep phase disorder (DSPD) is characterized by a persistent and intractable delay of sleep onset and offset time comparing to normal person, generally more than 2 h. People with DSPD are unable to fall asleep and wake up at socially acceptable times, resulting in excessive daytime sleepiness (Micic, Lovato et al., 2016). According to a large population-based study with 10,220 adolescents aged 16–18 years conducted in Hordaland County of Norway, the prevalence of DSPD in the general population is estimated to 3.3%, and significantly higher among girls (3.7%) than boys (2.7%) (Sivertsen, Pallesen et al., 2013).

DSPD also has a strong heredity and familial tendency (Barclay, Eley et al., 2010; Koskenvuo, Hublin et al., 2007). In 2001, Ancoli-Israel et al. reported a DSPD pedigree with a bilineal mode of inheritance, as both the paternal and maternal branches contained affected individuals (Ancoli-Israel, Schnierow et al., 2001). Per3 is the first gene to be associated with DSPD. Ebisawas et al. identified six variants in hPer3 in DSPD individuals, and one haplotype is found to be significantly associated with DSPD (Ebisawa, Uchiyama et al., 2001). Furthermore, the contribution of a variable-number tandem-repeat polymorphism in the coding region of PER3 to extreme diurnal preference (ASPD or DSPD) is also investigated (Table 1). Archer et al. demonstrated that the shorter allele (PER3(4/4)) is strongly associated with DSPD. Consistently, homozygous Per3 knockout mice display a free-running period of 30 min shorter than the WT mice (Zhang, Hirano et al., 2016).

Recently, Patke et al. report a missense mutation (c.1657+3A > C) in hCry1 as a causative factor in a DSPD pedigree (Table 1) (Patke, Murphy et al., 2017). This mutation disrupts the splicing recognition site before exon11, resulting in the deletion of exon 11 with an in-frame deletion of 24 amino acids of CRY1 (CRY1Δ11). The CRY1Δ11 shows enhanced inhibition on CLOCK-BMAL1 heterodimer. This gain-of-function CRY1 variant causes reduced expression of a variety CLOCK-BMAL1 targets and lengthens the period of circadian molecular rhythms. Intriguingly, CRY1Δ11 mutation has a frequency of up to 0.6% in the general Caucasian population, suggesting it may be responsible for the abnormal sleep patterns in a sizeable human population.

### The Adverse Consequences of Circadian Rhythm Sleep Disorders

It is well known that disrupted circadian rhythms are associated with a variety of diseases, such as cancer, mental disorders, and metabolic disorders (Bass & Lazar, 2016; Ruan, Yuan et al., 2021). Studies on human with familial CRSD or corresponding genetically modified animal models provide further insight into this connection.

Xu and colleagues take advantage of S622G-PER2 transgenic mice, which mimic human ASPD, to investigate the effect of disrupted circadian clock on cell cycle progression and tumorigenesis. Their found that the X-ray induced apoptosis was markedly attenuated in cells from PER2-S662G:Per2−/− mice as compared with those from the control mice. And PER2-S662G mutation leads to an increased E1A- and RAS-mediated oncogenic transformation. In addition, the expression profiles of p21 and Cyclin D, two clock-controlled cell cycle genes, change significantly in the embryonic fibroblast cells taken from PER2-S662 mutant mice. These findings suggest that the ASPD-associated PER2-S662G mutation may enhance tumorigenesis (Gu, Xing et al., 2012).

Several studies reported that individuals with CRSD accompany with some neuropsychiatric symptoms. For instance, the individuals carrying the CKIδ-T44A mutation, show ASPD as well as migraine (Brennan, Bates et al., 2013). And individuals carrying the CRY1Δ11 variant show a combination of DSPD and attention deficit/hyperactivity disorder (ADHD) (Onat et al., 2020). Nevertheless, the mechanisms how these mutations lead to neuropsychiatric symptoms are still elusive.

Individuals carrying PER3-P415A/H417R show ASPD accompanied by higher Beck Depression Inventory and seasonality scores. Consistently, hPER3-P415A/H417R transgenic mice also show a mild depression-like phenotype, and Per3 knockout mice also present with depression-like behavior, suggesting a role for PER3 in mood regulation (Zhang et al., 2016).

## Conclusion and Perspective

Great advances have been made in the genetic basis of circadian rhythm sleep disorder in the past 20 years. These human genetic studies not only accelerate the understanding the mechanisms underlying circadian regulation, but also provide great opportunity to understand the connection between disrupted circadian rhythms and human health. However, most studies focus on the genetics of ASPD and DSPD, whereas few gene mutation was characterized on the irregular sleep-wake rhythm disorder and the non-24-h sleep-wake rhythm disorder although these disorders may also have a genetic component. It also should be noted that most CRSD-related genetics studies are based on rare and specious pedigrees. Nowadays, the biobanks, such as United Kingdom Biobank, which deposit both genetic and phenotypic information for huge number of individuals are available. One can predict that population-based whole genome-wide genetic analysis and/or cross-phenotypic analysis will greatly improve our understanding on the genetic basis of CRSDs and their consequences on the human health.

## Data Availability

The original contributions presented in the study are included in the article/Supplementary Material, further inquiries can be directed to the corresponding authors.
